# Enhanced Thermoelectric Properties of Ca_3−x_Ag_x_Co_4_O_9_ by the Sol–Gel Method with Spontaneous Combustion and Cold Isostatic Pressing

**DOI:** 10.3390/ma11122573

**Published:** 2018-12-17

**Authors:** Youyu Fan, Xiaoling Qi, Dechang Zeng

**Affiliations:** 1 School of Materials Science and Engineering, South China University of Technology, Guangzhou 510640, China; fanyouyu@163.com; 2 Guangzhou Institute of Energy Conversion, Chinese Academy of Sciences, Guangzhou 510640, China; qixl@ms.giec.ac.cn

**Keywords:** cobaltite ceramic, doping, thermoelectric properties, sol–gel method, cold isostatic pressing

## Abstract

In this study, Ca_3−x_Ag_x_Co_4_O_9_ ceramics were synthesized by the sol–gel method combined with spontaneous combustion and cold isostatic pressing. The Ca_3−x_Ag_x_Co_4_O_9_ ceramics were characterized via X-ray diffraction and scanning electron microscopy. Thermoelectric properties of the ceramics were measured from 323 to 673 K. The results indicated that Ag doping significantly affected the microstructure and thermoelectric properties. With the increase in Ag content and gradual increase in electrical conductivity, the Seebeck coefficient first increased and then decreased, whereas the thermal conductivity exhibited the opposite case. The figure of merit, *ZT*, was 0.17 at 673 K for the Ca_2.8_Ag_0.2_Co_4_O_9_ sample. These results indicated that the thermoelectric properties could be optimized remarkably with the substitution of Ag.

## 1. Introduction

Many industrial production processes or similar sources of significant waste heat have the characteristics of being dispersed and small scale. It is difficult to recover and eventually discharges direct waste heat, resulting in numerous heat source waste and major environmental pollution. Thermoelectric materials are a type of new promising energy conversion materials, which provide a better solution for solving the problem of the direct discharge of waste heat [1]. Direct conversion between heat and electricity can be realized. Thermoelectric conversion does not require traditional mechanical transmission parts and does not produce discarded objects during processing. This type of promising energy conversion material does not generate noise and causes no pollution to the environment.

Excellent thermoelectric materials should have a higher thermoelectric performance, which is expressed by *ZT*, where *ZT = S^2^σT/κ*, in which *S* is the Seebeck coefficient, *σ* is the electrical conductivity, *κ* is the thermal conductivity, and *T* is the absolute temperature. According to previous studies, alloy thermoelectric materials have higher thermoelectric performance normally. For example, Eden Hazan et al. [2] reported that the *ZT* of 0.67% Cu-doped (GeTe)_0.95_(BiTe) _0.05_ at 633 K is about 1.65. However, compared to alloy thermoelectric materials, oxide thermoelectric materials have many advantages. For example, oxide thermoelectric materials have better stability and anti-oxidation at high temperatures with a long service life and simple preparation, which provides good application prospects in high-temperature waste-heat power generation. Since Ca_3_Co_4_O_9_ was found to exhibit good thermoelectric properties by Masset et al. [3], it has been widely studied. The structure and performance of Ca_3_Co_4_O_9_ are more stable than those of Na_x_CoO_2_ at high temperatures because of its stable insulating layer structure, which makes Ca_3_Co_4_O_9_ more suitable for the direct conversion of high-temperature waste heat into electric energy. However, the poor thermoelectric properties of oxide thermoelectric materials are the principal obstacles in their widespread application. Identifying a method to obtain Ca_3_Co_4_O_9_ ceramics with high thermoelectric properties has become important in recent years [4,5,6,7].

Higher thermoelectric properties can be obtained by optimizing the preparation process and element doping. Kenfaui et al. [8] obtained Ca_3_Co_4_O_9_ by Spark Plasma Sintering (SPS), in which the power factor was 315 uW/mK^2^ at 840 K. Liu et al. [9] obtained the thermoelectric material Ca_3_Co_4_O_9_ via discharge plasma sintering. Kwon et al. [10] produced Ca_3_Co_4_O_9_ with an ideal *ZT* value by using a multi-layer sintering with SPS. Butt et al. [11] used SPS and dual doping by La and Fe to obtain the thermoelectric material Ca_3_Co_4_O_9_. Wu et al. [12] explored the effects of Y and Fe co-doping on the high-temperature thermoelectric properties of the thermoelectric material Ca_3_Co_4_O_9_ prepared by the combustion method. Constantinescu et al. [13] showed that Ca substitution by Sr significantly improved the electrical conductivity of Ca_3_Co_4_O_9_, which caused a 50% increase in the power factor relative to the un-doped Ca_3_Co_4_O_9_ at 1073 K.

In this study, Ca_3−x_Ag_x_Co_4_O_9_ thermoelectric materials were fabricated by the sol–gel process combined with spontaneous combustion and cold isostatic pressing. Moreover, Ag was used as the doping element. Through Ag doping on the Ca site, the mechanism of its effect on the thermoelectric performance of Ca_3_Co_4_O_9_ was studied.

## 2. Experiment

### 2.1. Synthesis of Ca_3−x_Ag_x_Co_4_O_9_

High-purity Ca(NO_3_)_2_·4H_2_O and Co(NO_3_)_2_·6H_2_O as the original materials and pure AgNO_3_ for doping were dissolved completely in an absolute ethyl alcohol solution of citrate acid. To prepare this solution, citrate acid was dissolved in ethanol in the mole ratio *n*(COOH^−^)/*n*(NO_3_^−^) of 0.8. AgNO_3_ was added in different mole ratios (x = 0.05, 0.1, 0.2, 0.3, and 0.5) according to chemical formula Ca_3−x_Ag_x_Co_4_O_9_. Polyethylene glycol (PEG) 800 was added as the dispersant with a mass ratio (wt%) of 4%. Uniform and transparent precursor solutions were obtained by magnetic stirring, which were heated at 353 K with continuous stirring in a constant-temperature water bath to form wet gels. The wet gels were dried in a drying cabinet to obtain porous dry gels. After spontaneous combustion, the dry gels were sintered at 1023 K for 2 h in a muffle furnace to obtain the precursor powder. The precursor powder was pressed under a pressure of 2 MPa for 15 s and then subjected to cold isostatic pressing under a pressure of 200 MPa for 2 min. Next, the samples were sintered at 1073 K for 18 h in a muffle furnace.

### 2.2. Characterization and Measurements

The crystal structure of the samples was studied via X-ray diffraction (XRD) on a Philips Model X’Pert PRO diffractometer (Philips, Almelo, Netherlands) in the range of 5–80°, using CuKα radiation with voltage of 40 kV and current of 40 mA. The microstructure was characterized by scanning electron microscopy (SEM, XL30FEG, Philips, Eindhoven, Netherlands) with a voltage of 10 kV. The Seebeck coefficient and electrical conductivity were determined using ULVAC-RIKO ZEM3 (ULVAC, Kanagawa, Japan); the sintered samples were cut into rectangular bar specimens of 15 × 3 × 2 mm^3^ in size and measured from 323 K to 673 K with temperature gradients of 20 K, 30 K and 40 K. The thermal conductivity was obtained from the thermal diffusivity (*α*) and the specific heat capacity (*C_p_*) was tested by the laser flash method (Netzsch Geratebau GmbH LFA447, NETZSCH, Selb, Germany) and Differential Scanning calorimeter (Differential Scanning Calorimeter 204F1, NETZSCH, Selb, Germany). The thermal conductivity (*κ*) was derived according to the formula, *κ* = *αC_p_ρ,* the experimental density (*ρ*) can be measured by the Archimedes method. The sintered samples were cut into circular sheet specimens of Φ12 mm × (1.5–2) mm in size for thermal conductivity testing. The theoretical density is calculated by formula (mass = volume density).

## 3. Results and Discussion

### 3.1. Analysis of XRD Patterns of Ca_3−x_Ag_x_Co_4_O_9_

**Figure 1:**
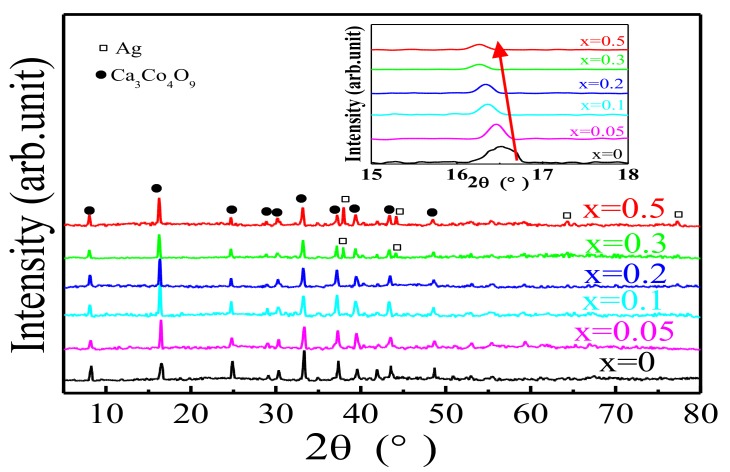
The X-ray diffraction (XRD) patterns of the Ca_3__−x_Ag_x_Co_4_O_9_ (x = 0–0.5) samples.

Figure 1 presents the XRD patterns of the Ca_3−x_Ag_x_Co_4_O_9_ XRD (x = 0–0.5) samples. Except for the diffraction peak of Ag (No. 04-0783), those of all the samples correspond to Ca_3_Co_4_O_9_ (No. 23-0110) of the JCPDS standard card. With increasing Ag doping amount, the positions of the diffraction peaks of the sample are gradually shifted by a small angle (the inset shows the 2*θ* shifts of the (002) diffraction peak). When the amount of doping is x ≤ 0.2, Ag completely enters the Ca_3_Co_4_O_9_ lattice but does not change the corresponding structure. When x = 0.3 or 0.5, diffraction peaks of Ag appear in the XRD patterns, and the diffraction peak intensity of Ag increases with increasing Ag addition. This is probably because a large amount of Ag cannot completely replace Ca in the lattice and exists in the form of an Ag substance in the samples.

### 3.2. Analysis of Cell Parameters

Based on the diffraction data of the samples, the cell parameters of Ca_3−x_Ag_x_Co_4_O_9_ (x = 0–0.5) are calculated and listed in Table 1. With increasing Ag doping, cell parameters *a*, *b1*, and *c* increase simultaneously. Because the radius of Ag^+^ is 1.26 Å and that of Ca^2+^ is 0.99 Å, the ionic radius ratio of Ag^+^ to Ca^2+^ of 1.27 is large. When Ag^+^ enters the Ca_2_CoO_3_ layer of the Ca_3_Co_4_O_9_ lattice and replaces the Ca site, a, b1, and c increase simultaneously due to lattice distortion. Moreover, a larger doping amount of Ag implies a more significant effect on the structure of the Ca_3_Co_4_O_9_ crystal. Therefore, the cell volume of Ca_3−x_Ag_x_Co_4_O_9_ (x = 0–0.5) increases with the increase in Ag doping, even if the change is not significant. The theoretical density increases with increasing Ag doping, as exhibited in Table 1. There are two possible reasons for the density increase. One is that Ag contributes to the heavier Ca_3_Co_4_O_9_, and the other is that the cold isostatic pressing method improves the porosity of the samples. The experimental density in the Table 1 was achieved by the Archimedes method with five samples for each group. According to the results, experimental density was increased with the increase of doping Ag contents, and the standard deviation is 0.02–0.03 for all groups.

### 3.3. SEM Analysis 

**Figure 2:**
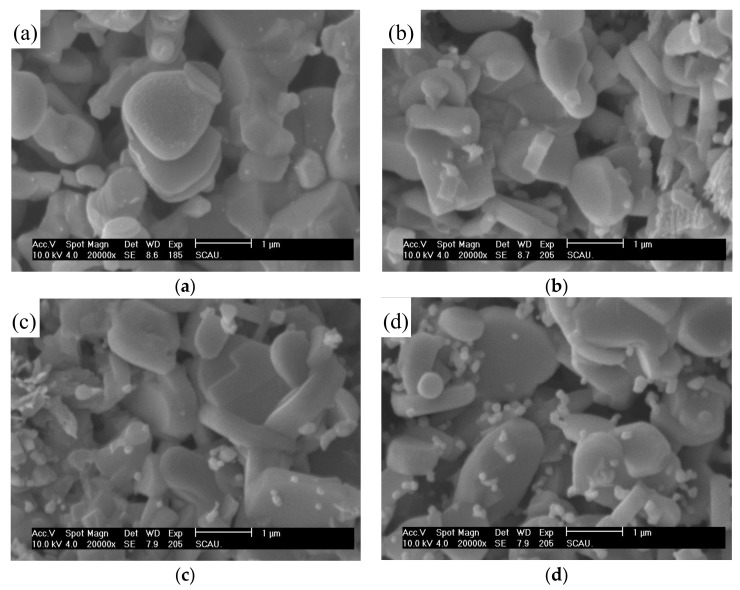
The scanning electron microscopy (SEM) micrographs of the Ca_3−x_Ag_x_Co_4_O_9_ samples. (**a**) x = 0.05; (**b**) x = 0.2; (**c**) x = 0.3; (**d**) x = 0.5.

Figure 2 displays the SEM micrographs of the Ca_3−x_Ag_x_Co_4_O_9_ (x = 0–0.5) samples. It can be observed that the particle sizes of the samples increase slightly with increasing Ag content. This is owing to the substitution of Ca^2+^ in the Ca_3_Co_4_O_9_ lattice by Ag^+^, which causes lattice distortion and increases the cell volume. When doping amount x = 0.3, some secondary particles are observed. With increasing doping amount (x = 0.5), the content of the secondary phase particles increases. Based on the XRD analysis, the second phase particles are Ag particles segregated from the Ca_3_Co_4_O_9_ lattice.

### 3.4. Electrical Conductivity

**Figure 3:**
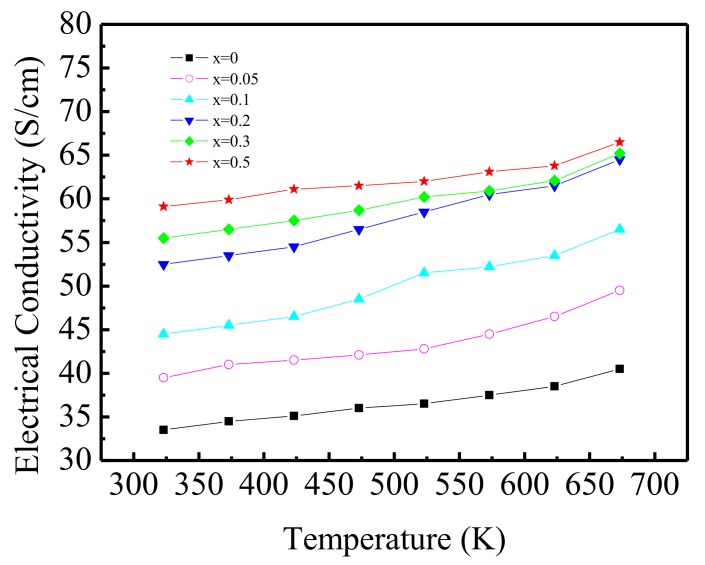
Temperature dependence of electrical conductivity of Ca_3__−x_Ag_x_Co_4_O_9_ (x = 0–0.5) samples.

Figure 3 presents the *σ–T* behaviour of the Ca_3−x_Ag_x_Co_4_O_9_ (x = 0–0.5) samples. The electrical conductivity increases with increasing temperature in the temperature range measured, indicating a transmission characteristic similar to that of a semiconductor. With the increase in Ag, there is an obvious electrical conductivity increase from 38.5 S cm^−1^ (x = 0) to 66.2 S cm^−1^ (x = 0.5) at 673 K.

The carrier mobility and carrier concentration are two key factors affecting the electrical conductivity. Grain boundary scattering, lattice vibration scattering and other factors affect the carrier mobility of a semiconductor. More scattering implies a lower carrier mobility. The conduction of Ca_3_Co_4_O_9_ is followed by hole conduction mechanism. When a Ag^+^ ion enters the Ca_2_CoO_3_ layer for replacing Ca^2+^, more holes are generated to maintain the balance of the valence state, which increases the concentration of holes. Therefore, the electrical conductivity of the material increases with the increase in the Ag doping amount. When the amount of Ag doped increases sufficiently, simple Ag substances appear and accumulate on the surface of the material grains, which can weaken the carrier scattering and decrease the resistance on the grain boundary. In addition, the resistivity of Ag is very low. Therefore, these two reasons cause the conductivity of Ca_3−x_Ag_x_Co_4_O_9_ (x = 0–0.5) to increase with the increase in Ag doping.

**Figure 4:**
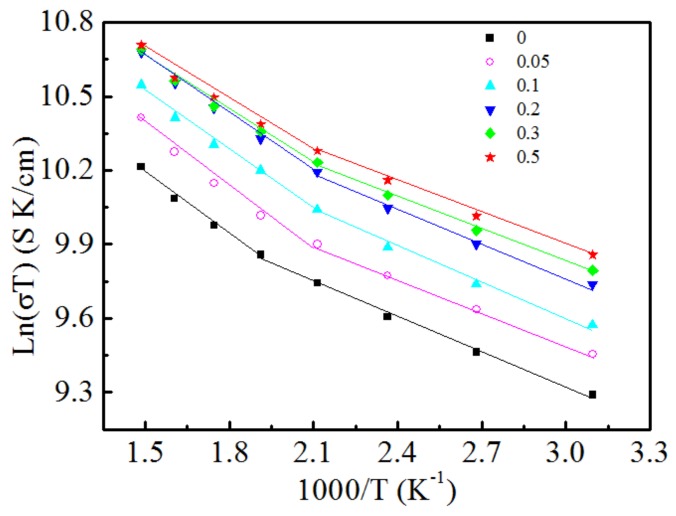
ln(σT) *versus* 1/T of Ca_3−x_Ag_x_Co_4_O_9_(x = 0–0.5) samples.

The diagram of the relationship between ln(*σT)* and *1/T* of the Ca_3-x_Ag_x_Co_4_O_9_ (x = 0–0.5) samples is presented in Figure 4. All samples exhibit a drift conductance characteristic above 470 K. The activation energies of the Ca_3−x_Ag_x_Co_4_O_9_ (x = 0–0.2) samples are practically similar, which indicates that the replacement of Ca^2+^ by Ag^+^ does not change the transmission process of the material and transmission mechanism (hole). The substitution of Ag^+^ for Ca^2+^ occurs in the insulating layer Ca_2_CoO_3_ and does not enter the conductive layer of CoO_2_; therefore, the transmission pathway does not get destroyed and activation energy does not change. When x > 0.2, the activation energy of the samples slightly decreases, which indicates a change in the material transfer mechanism. This may be due to the accumulation of Ag on the grain surface of the material, which provides an electronic conducting mechanism and makes the transport property of the system complex.

### 3.5. Seebeck Coefficient

**Figure 5:**
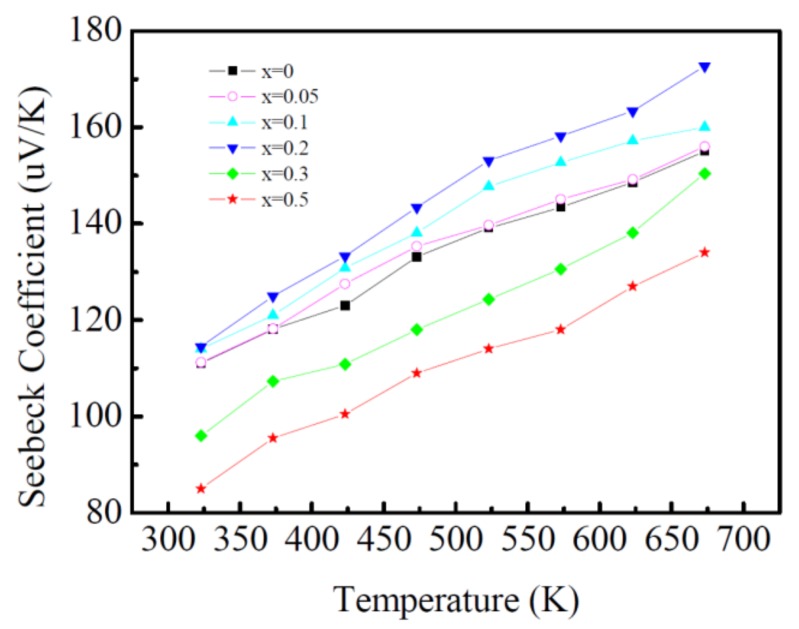
Temperature dependence of Seebeck coefficient of Ca_3−x_Ag_x_Co_4_O_9_ (x = 0–0.5) samples.

Figure 5 displays the temperature dependence of the Seebeck coefficient of the Ca_3−x_Ag_x_Co_4_O_9_ (x = 0–0.5) samples. Positive Seebeck coefficients, which indicate hole conduction, increase monotonically with the temperature increase. Ag doping does not change the conductivity type of the materials. When the amount of Ag doping is less than 0.3, the Seebeck coefficient increases with the increasing Ag doping amount. The Seebeck coefficient of Ca_2.8_Ag_0.2_Co_4_O_9_ is maximum at 673 K with 172.7 μV K^−1^, which is similar to the research results of other Ca_3_Co_4_O_9_/Ag systems reported by Wang et al. [14,15] and the NaCo_2_O_4_/Ag system by Seetawan et al. [16,17], and also similar in value to that of Ca_2.93_Ag_0.072_Co_4_O_9_, which was fabricated by Sun et al. [18] via pulsed laser deposition. When the Ag doping amount is 0.3, the Seebeck coefficient is lower than that of the un-doped sample. When the content of Ag reaches 0.5, the Seebeck coefficient is at the minimum level.

According to the semiconductor theory [19], the relationship between the Seebeck coefficient and carrier concentration is a negative correlation. However, the Seebeck coefficient of Ca_3−x_Ag_x_Co_4_O_9_ increases with the increasing carrier concentration induced by Ag doping (when x ≤ 0.2). This cannot be explained by the traditional theory, and should be related to the strong correlation of the electron [20]. The mobility of the carrier should play an important role in the change in the Seebeck coefficient in this material system. The Seebeck coefficient can be expressed by Formula (1) [21].

(1) $$S\left(T\right)=\frac{c_{e}}{n}+\frac{\pi^{2}k_{B}^{2}T}{}{\left[\frac{\partial \ln\mu \left(\varepsilon \right)}{\partial \varepsilon }\right]}_{\varepsilon =E_{F}}$$

where, *n* is the carrier concentration;

μ(ε) is the carrier mobility;
*C_e_* is the heat capacity;
*K_B_* is the Boltzmann coefficient.

At a certain temperature, *S* depends on the carrier concentration and carrier mobility. The substitution of Ag^+^ for Ca^2+^ can increase the carrier concentration and change the mobility of the carrier. Although the carrier concentration is inversely proportional in the first part of Formula (1), the experimental results show that the Seebeck coefficient increases with the increase in Ag doping, it is concluded that the change in the carrier mobility is the main cause for the increase in the Seebeck coefficient with the content of Ag (x ≤ 0.2).

When the Ag doping amount continues to increase (x > 0.2), the redundant Ag^+^ cannot completely replace Ca^2+^ and exist in the form of an Ag substance. It amounts to a two-phase system (Ca_3−x_Ag_x_Co_4_O_9_ and Ag), and the Seebeck coefficient of the system can be expressed as Formula (2) [15]:

(2) $$S=\underset{i}{\sum }\left(\frac{\sigma_{i}}{\sigma }\right)S_{i}$$

The conduction mechanism of the Ag substance is via electrons, and the Seebeck coefficient is negative and low [22] (32 uV K^−1^ at 373K), which has an obvious weakening effect on the Seebeck coefficient of the Ca_3−x_Ag_x_Co_4_O_9_ system. Therefore, the Seebeck coefficient decreases significantly with the increase in Ag doping (x > 0.2).

### 3.6. Thermal Conductivity

**Figure 6:**
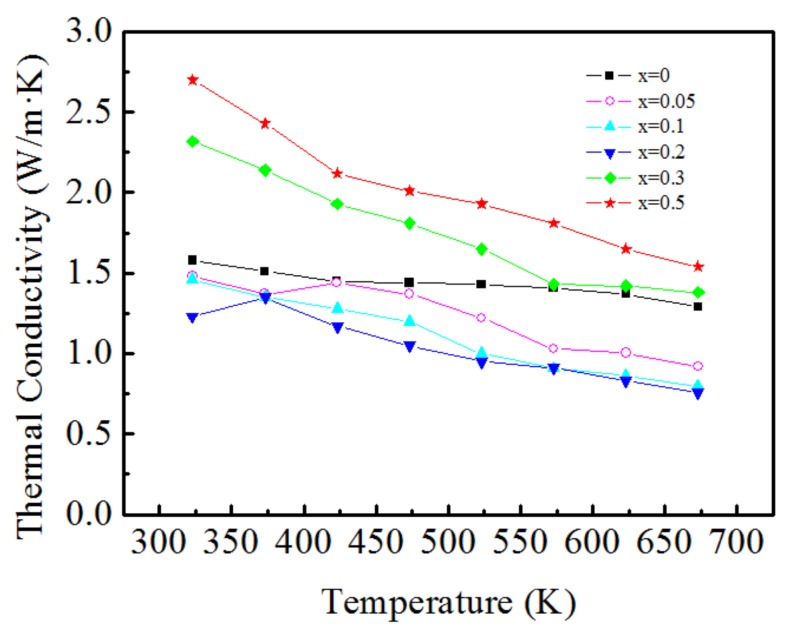
Temperature dependence of the thermal conductivity of Ca_3−x_Ag_x_Co_4_O_9_ (x = 0–0.5) samples.

The temperature dependence of the thermal conductivity of the Ca_3−x_Ag_x_Co_4_O_9_ (x = 0–0.5) samples is shown in Figure 6. The thermal conductivities of all the doped samples decrease with the increase in the temperature. When x ≤ 0.2, the doped samples exhibit a lower thermal conductivity than the un-doped Ca_3_Co_4_O_9_, which indicates that Ag doping at the Ca site can decrease the total thermal conductivity. This shows that the doping is beneficial for reducing the thermal conductivity. The lowest thermal conductivity is 0.76 W/mK at 673 K for the sample with x = 0.2. When the doping content exceeds 0.2, the total thermal conductivity increases and is even larger than that of the un-doped sample.

Typically, the carrier concentration can vary over a wide range for semiconductor materials. The thermal conductivity is dominated by phonon vibrations when the semiconductor material has a low carrier concentration. However, it is dominated by the concentration of the carriers when the carrier concentration increases. Therefore, in this study, when x ≤ 0.2, the phonon vibrations are the key factor. Because Ag substitutes Ca in the system, defects and impurities are formed, causing crystal distortion, which strengthens the phonon scattering. Therefore, phonon scattering shortens the mean free path of the phonon layer and decreases κ_l_. Therefore, with more Ag doping, the total thermal conductivity reduces. As for x > 0.2, the thermal conductivity of the samples shows different characteristics from x ≤ 0.2. One possible explanation for this is due to Ag particles turned up with excessive Ag doping, as shown in Figure 2. The thermal conductivity of Ag is 4.29 W cm^−1^ K^−1^, which is much higher than that of Ca_3_Co_4_O_9_, causing the thermal conductivity to become higher than those of the less-doped samples. On the other hand, according to the relative density in Table 1, the relative density of the samples increases when x > 0.2, which indicates that the decrease in oxygen vacancies will lengthen the mean free path of the phonons, so that the thermal conductivity increases. This phenomenon is also found in Sr and Yb co-doped CaMnO_3_ [23]. Overall, the combination of these two factors results in a higher thermal conductivity that is even much higher than that of the un-doped samples when x > 0.2.

**Figure 7:**
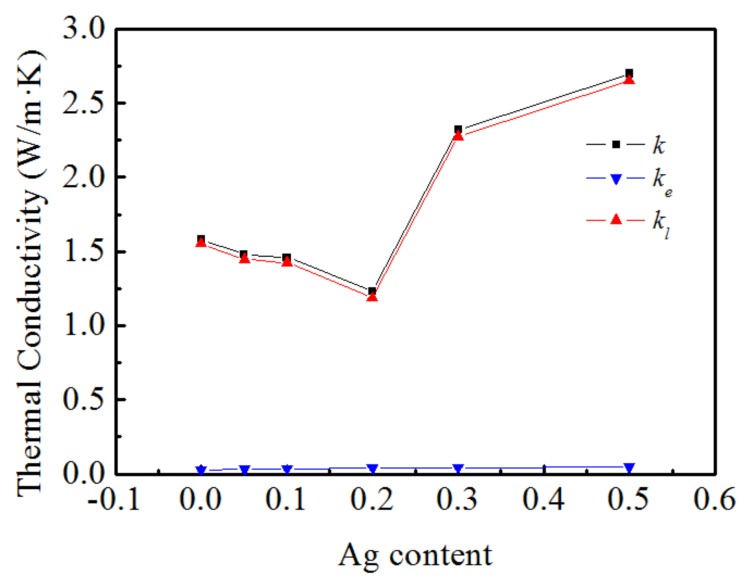
Ag content dependence of thermal conductivity *κ*, electronic thermal conductivity *κ_e_* and lattice thermal conductivity *κ_l_* for Ca_3−x_Ag_x_Co_4_O_9_ samples at room temperature.

Thermal conductivity (*κ)* is constituted by lattice thermal conductivity *κ_l_* and electron thermal conductivity *κ_e_* can be expressed as *κ* = *κ_l_* + *κ_e_*. *κ_e_* can be calculated by Wiedemann-Franz law [24] as *κ_e_* = *L_0_σT*, where *L_0_, σ, T* are Lorenz number *L_0_* = 2.45 × 10^−8^ W·Ω·K^−2^, measured electrical conductivity and absolute temperature respectively. Figure 7 presents the variation in the thermal conductivity, namely, thermal conductivity of the phonons and electron thermal conductivity with Ag doping at 323 K. It can be seen that the thermal conductivity and lattice thermal conductivity first decrease and then increase with the increase in the Ag doping amount, and the change range is consistent. However, the *κ_e_* values were from 0.027 W/mK to 0.047 W/mK for Ag doping from 0 to 0.5 at 323 K; the electronic thermal conductivity barely varies with the increase in the doping amount. The above analysis further shows that the thermal conductivity of the material is mainly determined by the thermal conductivity of the lattice and that the electronic thermal conductivity has little effect on the total thermal conductivity.

### 3.7. Thermoelectric Figure-of-Merit

**Figure 8:**
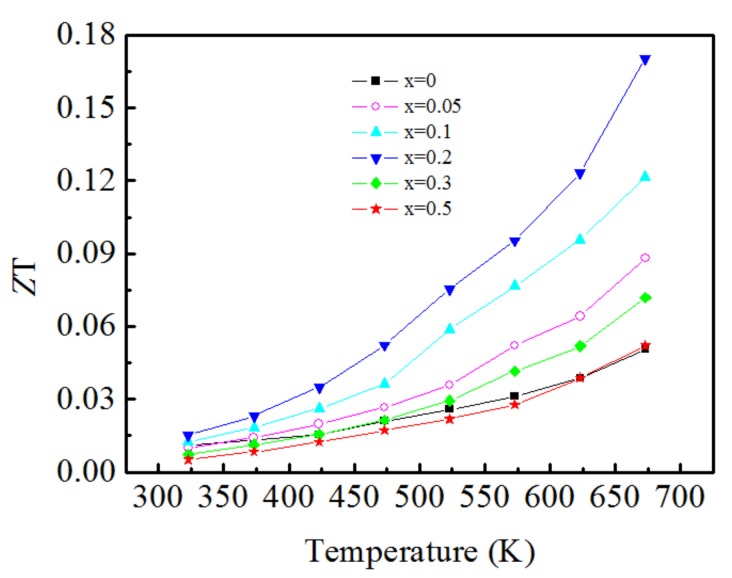
Temperature dependence of figure of merit of Ca_3−x_Ag_x_Co_4_O_9_(x = 0–0.5) samples.

The temperature dependence of the dimensionless figure of merit *ZT* of the Ca_3−x_Ag_x_Co_4_O_9_ samples is displayed in Figure 8. The *ZT* values of all samples increase with increasing temperature in the entire test temperature range. The higher the temperature, the larger the increase is. This indicates that the Ca_3−x_Ag_x_Co_4_O_9_ material is suitable for use in a high-temperature field. An appropriate amount of Ag doping is beneficial for enhancing the figure of merit, and the *ZT* values increase with increasing doping amount up to x = 0.2. When x > 0.2, the *ZT* values decrease significantly with increasing Ag-doping content. The highest *ZT* value is obtained for the Ca_2.8_Ag_0.2_Co_4_O_9_ sample, which reaches 0.17 at 673 K, which was 0.07 higher than that of Ca_2.9_Ag_0.1_Co_4_O_9_, which was synthesized by Feipeng et al. [25], and 0.09 higher than the result of Yin et al. [26].

## 4. Conclusions

In this study, Ca_3−X_Ag_X_Co_4_O_9_ were fabricated as thermoelectric materials by the sol–gel method combined with spontaneous combustion and cold isostatic pressure sintering. This fabrication method exhibited the advantages of high particle purity, good dispersity, good grain shape, and low production cost. Ag doping has significant effects on the microstructure and thermoelectric properties. Increasing the Ag doping increases the electrical conductivity and Seebeck coefficient, but it is unfavourable for reducing the thermal conductivity. An appropriate Ag doping content improves the thermoelectric properties, and the highest *ZT* of 0.17 at 673 K is obtained in the Ca_2.8_Ag_0.2_Co_4_O_9_ sample, for which the value is over three times higher than that of un-doped Ca_3_Co_4_O_9_.

## Figures and Tables

**Table 1:** Lattice parameters *a*, *b*_1_, and *c*, unit-cell volume V, and density of Ca_3−x_Ag_x_Co_4_O_9_ polycrystalline samples.

Samples	*a* (Å)	*b*_1_ (Å)	*c* (Å)	*V* (nm^3^)	Theoretical Density (g/cm^3^)	Experimental Density (g/cm^3^)	Relative Density (%)
Ca_3_Co_4_O_9_	4.8352	4.5531	10.8382	0.2386	4.24	3.900	91.98
Ca_2.95_Ag_0.05_Co_4_O_9_	4.8366	4.5532	10.8390	0.2387	4.25	3.902	91.82
Ca_2.9_Ag_0.1_Co_4_O_9_	4.8399	4.5589	10.8435	0.2393	4.27	3.914	91.66
Ca_2.8_Ag_0.2_Co_4_O_9_	4.8443	4.5632	10.8503	0.2399	4.30	3.930	91.40
Ca_2.7_Ag_0.3_Co_4_O_9_	4.8446	4.5673	10.8552	0.2402	4.33	3.980	91.92
Ca_2.5_Ag_0.5_Co_4_O_9_	4.8448	4.5701	10.8584	0.2404	4.41	4.120	93.42
